# Medical Decision Making: The Family–Doctor–Patient Triad

**DOI:** 10.1371/journal.pmed.0020129

**Published:** 2005-06-28

**Authors:** Fawad Aslam, Omar Aftab, Naveed Z Janjua

**Affiliations:** **1**Aga Khan University Medical College, Karachi, Pakistan; **2**Aga Khan University, Karachi, Pakistan

The importance of a person-centred approach and the intricacies of risk communication have recently been well described in *PLoS Medicine* [1,2]. The applicability of the patient-centred approach to Eastern countries, however, has cultural, religious, and practical impediments that demand careful consideration. The bulk of the world population lives outside the United States and western Europe. Unlike in the West where the patient takes centre stage by both tradition and law, the family–doctor–patient triad is the norm in Eastern states, in general, and Pakistan in particular [3–8].

Pakistan is a predominantly Muslim country of 150 million people. About half the population is uneducated, and more than a third lives below the poverty line. There is one doctor for every 1,432 patients, compared to one doctor for every 390 patients in the US. The health-insurance system is virtually nonexistent, and there is no concept of assisted-care living, with the care of the elderly largely taking place at homes by their families. Strongly held religious beliefs and cultural views govern everyday life and dictate the roles of every member of the society. Families consist of well-knit, supportive, and collectively earning interdependent members who take mutual decisions on all matters pertaining to life and death [3,4]. The elder members of the family command the greatest respect and authority. The family unit is the functional unit of the society, the dynamics of which need attention and respect.

Strong family systems and the authoritative position of the doctor are the governing forces of medical decision making in these countries. Illiteracy, poverty, poor awareness of patient's rights, and a lack of accountability for physicians are factors conducive to such a practice. With this background, the role of the patient is limited. Health expenditure is borne by the family, giving it a central role in decision making. The concept of the financial survival of the family is a harsh reality [3,4]. The health-care costs of one seriously ill member may jeopardize the survival of others by draining the limited resources. Due to familial, moral, and monetary support, the patient relinquishes the responsibility of decision making and gives the primary role to the family or the doctor. Women, for example, may not give consent unless they get approval from their spouses [5]. In the case of women, who may have a lesser say in the patriarchal family system, the doctor should strive for active participation. Sometimes the family aims to protect the patient from stress by withholding information, and in terminal illnesses, the doctor and family act in concert to conceal complete information from the patient. They, for example, may not mention the word cancer to patients who have malignancies [9].

In contrast to Western practice, the role of the doctor is authoritative. Doctors are regarded as instruments of God and given the final authority in decision making [3,4,10]. In such circumstances, doctors are likely to take decisions unilaterally. When they do involve patients in decision making, physicians accept the centrality of families, with some considering patients and families as one [5]. One worry regarding communication of harm is of losing patients to other physicians with a more reassuring “nothing will go wrong” attitude [5]. It is also said that more time and patience are required to explain things to the illiterate. It is perhaps impractical, therefore, to expect overworked and underpaid physicians to practice risk communication according to the book.

Thus, the concept of individual centrality that is so elementary in the West stands challenged in the East. Research is needed to formulate appropriate strategies of risk communication. Areas needing research include the patient's concept of autonomy, the role of the family as perceived by patients and doctors, the existing practices of medical decision making, and the training of doctors in communicating risk.

An economically sound and literate population, properly trained doctors, and institutional checks and balances are essential prerequisites for establishing decision making with parity of partners. The need is to find a middle ground where not only the family unit is respected, but the patient also plays a proactive role. A dynamic balance between cultural values of caring and the possibility of a more individualistic role in health care is needed and is, indeed, attainable. Doctors, being the most influential component of the family–doctor–patient triad, can play a significant role in bringing about this change.
